# Impact of Moriamin Forte on Testicular and Epididymal Damage in Rats with Oligoasthenospermia

**DOI:** 10.1155/2021/4059248

**Published:** 2021-06-09

**Authors:** Xing Zhou, Guo-Wei Zhang, Wei-Ning Liang, Yi-Ze Li, Song Xu, Ling-Ling Yan, Hong-Jun Li, Xue-Jun Shang

**Affiliations:** ^1^Department of Andrology, Jinling Hospital Affiliated to Nanjing University School of Medicine, No. 305, East Zhongshan Road, Nanjing, Jiangsu 210002, China; ^2^Department of Andrology, The First Affiliated Hospital of Hunan University of Chinese Medicine, No. 95, Shaoshan Middle Road, Changsha, Hunan 410007, China; ^3^Department of Urology, Suqian First Hospital, No. 120, Suzhi Road, Suqian, Jiangsu 223800, China; ^4^Department of Andrology, Jinling Hospital Affiliated to Southern Medical University, No. 305, East Zhongshan Road, Nanjing, Jiangsu 210002, China; ^5^Department of Urology, General Hospital of Eastern Theater Command, No. 305, East Zhongshan Road, Nanjing, Jiangsu 210002, China; ^6^Laboratory of Reproductive Medicine, Shenzhen Wanhe Enterprise Technology Center, 12/F, Block C, Wanhe Technology Complex, 7 Huitong Road, Guangming District, Shenzhen, Guangdong 518107, China; ^7^Department of Urology, Peking Union Medical College Hospital, Peking Union Medical College, Chinese Academy of Medical Sciences, No. 1 Shuaifuyuan, Dongcheng District, Beijing 100730, China

## Abstract

To investigate the effect and mechanism of action of Moriamin Forte (MF) on oligoasthenospermia (OA) in rats exposed to multiglycosides of *Tripterygium wilfordii* (GTW), forty male Sprague Dawley rats were randomly divided into four groups. Rats in the control group were treated with 0.5% sodium carboxymethyl cellulose. The remaining rats were administered GTW (30 mg/kg/d) for 40 d to establish an OA model. Concurrently, the groups were treated with normal saline and low-dose (100 mg/kg/d) and high-dose (200 mg/kg/d) MF, respectively. After treatment, the number and motility of sperm cells were examined. Testicular and epididymal histomorphology changes were observed. Antioxidant indicators (SOD, CAT, MDA, TAC, and Nrf2) in testicular and epididymal tissues were detected. Apoptotic and antiapoptotic indicators (Bax and Bcl2 expression) in the testicular tissue were measured by immunohistochemistry. GTW decreased sperm count and motility, damaged testicular and epididymis tissues, impaired antioxidase activity, and increased tissue MDA levels. Meanwhile, GTW upregulated the expression of Bax and downregulated the expression of Bcl2. Western blot analysis demonstrated a decrease in the Nrf2 expression in the model group. Treatment with MF improved sperm count and motility, as well as inhibited the rate of apoptosis in the rat reproductive system. Moreover, MF improved the activity of antioxidants and increased the relative expression of the antioxidant pathway-related protein Nrf2. In conclusion, MF may reverse the GTW-induced OA by modulating the expression of apoptotic and antioxidant pathway-related proteins. This study may provide a pharmacological foundation for the use of MF in OA treatment.

## 1. Introduction

Infertility is recognised in a couple that is unable to conceive within a year despite frequent, unprotected intercourse [1]; it affects males and females equally [2]. Semen analysis findings in men failing to conceive often include a decreased number of spermatozoa (oligozoospermia), impaired sperm motility (asthenozoospermia), or both (oligoasthenospermia, OA). Idiopathic male infertility refers to cases of infertility without a clear cause. Evidence-based pharmacological interventions may help treat idiopathic infertility [3, 4].

Multiglycosides of *Tripterygium wilfordii* (GTW), also known as *T. wilfordii* polysaccharides, are fat-soluble active compounds extracted from the roots of plants that belong to the Celastraceae family [5]. GTW has been used to treat autoimmune diseases such as rheumatoid arthritis and glomerulonephritis; however, it has also been associated with reproductive system toxicity and epididymal sperm deformities, decreased sperm concentration and motility, and the formation of vacuoles in the sperm mitochondrial sheath [6]. GTW may cause spermatogenic tubule atrophy, damage the epithelial cells of spermatozoa, and reduce sperm production [7]; each of these effects is progressive and increases in magnitude over time [8]. The present study used GTW to establish the OA model in rats.

A Moriamin Forte (MF) capsule contains essential amino acids (EAAs) and vitamins A, B, C, D, and E (Table 1), also known as compound amino acids (CAAs). Amino acids are the basic unit of protein. EAAs cannot be synthesised endogenously and thus require exogenous supplementation. Amino acids and vitamins are used to prevent and treat exercise-induced fatigue, scurvy, rickets, beriberi disease, burns, and fractures [9–14], as well as malnutrition in pregnant or lactating women and in children [15, 16]. EAAs are raw materials for sperm nucleic acid synthesis. They are involved in sperm formation, maturation, motility, and capacitation and are contained in the seminal plasma, providing a suitable environment for sperm development [17, 18]. A previous study has examined the role of EAAs and vitamins A, B, C, D, and E in the reproductive system [19–33]; however, the associated mechanism remains unknown. This study aimed to explore the effects and mechanisms of MF against GTW-induced OA in a rat model to establish a foundation for further research on the use of MF in the treatment of OA. This study provides theoretical basis for the future use of MF in the treatment of OA in clinical practice.

## 2. Materials and Methods

GTW (approval no. Z31020415, batch no. 160303, Shanghai, China) and MF (approval no. H20000478, batch no. 160601, Shenzhen, China; ingredients are presented in Table 1) were purchased from Shanghai Fudan Fuhua Pharmaceutical Co., Ltd.

### 2.1. Animals

All procedures involving animals were approved by the Institutional Animal Care and Use Committee of Jinling Hospital, affiliated with Southern Medical University (Nanjing, Jiangsu, China, SYXK (M) 2012-0047). Food and water were changed daily and provided without restrictions. Cage bedding was changed every other day and maintained clean and dry. All animals were adapted to the laboratory environment (temperature in the range of 20–26°C), which involved a 12 h light-dark cycle, introduced 1 week before the experiment. Forty male Sprague Dawley rats weighing between 200 and 250 g were provided by and housed at the animal facility of the Department of Comparative Medicine of the General Hospital of the Eastern Theatre Command (Nanjing, Jiangsu, China). The OA model was established by the gavage of GTW once daily for 40 days at a dose of 30 mg/kg/d, as previously reported [34].

### 2.2. Experimental Groups, Treatment, and Sample Preparation

Forty male rats were randomly divided into four groups. In the control group, rats were treated with 0.5% sodium carboxymethyl cellulose (CMC-Na, Aladdin-e Co., C104985). GTW doses were dissolved in 1 ml of CMC-Na. In the remaining rats, GTW was administered during 40 consecutive days to create the OA model. Meanwhile, the rats were treated with normal saline and low-dose (100 mg/kg/d) and high-dose (200 mg/kg/d) MF, respectively. The daily MF dose was dissolved in 1 ml of normal saline. All drugs were given to the rats by gavage.

### 2.3. Viscera Index

Rats were anaesthetised with pentobarbital (50 mg/kg, intraperitoneal injection) and sacrificed by cervical dislocation 24 h after the last treatment. The testis and epididymis were weighed. The viscera index was then calculated as the viscera weight/body weight (g) × 100%.

### 2.4. Sperm Count and Motility

The left epididymal tail was harvested and washed with saline. The tissue was weighed and minced in an Eppendorf tube with 1 ml saline and subsequently incubated at 37°C for 30 min ahead of epididymal spermatozoa collection. The epididymal spermatozoa suspension was sampled, diluted with saline, and transferred to the haemocytometer counting chambers to determine sperm count and motility.

### 2.5. Histopathological Analysis

The left testis and caput epididymis were fixed in 4% paraformaldehyde for histopathological and immunohistochemical analyses. Fixed tissues were routinely processed with an automatic tissue processor and embedded in paraffin. Each paraffin block was cut into slices of 4 *μ*m thickness; sections were stained with haematoxylin and eosin, following established protocols, and examined under a light microscope (Nikon Eclipse TS100, Japan).

### 2.6. Immunohistochemical Staining of Bax/Bcl2

Testis sections fixed in paraffin were treated for 20 minutes with xylene, rehydration, antigen retrieval, and blocking. The sections were then incubated with the primary antibody for Bax or Bcl2 in phosphate buffer saline overnight at 4°C (Bax: Abcam, ab32503; Bcl2: Abcam, ab32124). Subsequently, secondary antibodies (streptavidin-HRP, Abcam, ab7403) were added, and sections were incubated for 20 min. Primary and secondary antibody concentrations were at the ratio of 1/250 and 1/500, respectively. Nuclear staining and section dehydration were performed, and the transparency of the slides was observed under an optical microscope at ×200 magnification. Immunohistochemical images were semiquantitatively examined using image analysis software Image-Pro Plus 6.0 (IPP; produced by Media Cybernetics Corporation, USA). Ten fields were randomly selected from each section to determine the mean integrated optical density of the positive products.

### 2.7. Oxidation and Antioxidant Index

The right-side testis and epididymis were weighed and homogenised in normal saline. The supernatant was collected by centrifugation over 15 min at 4000 × g at 4°C. Protein concentrations were determined using the bicinchoninic acid (BCA) protein assay kit (Angle Gene Bioengineering Co., Ltd., Nanjing, China), according to the manufacturer's instructions. The levels of SOD (ANG-SH-10012, Angle Gene Bioengineering Co., Ltd., Nanjing, China) and CAT (ANG-SH-10121, Angle Gene Bioengineering Co., Ltd., Nanjing, China) activity and MDA (ANG-SH-10111, Angle Gene Bioengineering Co., Ltd., Nanjing, China) and TAC (ANG-SH-21121, Angle Gene Bioengineering Co., Ltd., Nanjing, China) concentrations in the testis and epididymis were measured by commercially available kits. The levels of absorbance were determined at 450 nm, 240 nm, 532 nm, and 593 nm.

### 2.8. Western Blot Analysis of Nrf2

The prepared testicular and epididymal tissue supernatants were examined. Protein concentration was measured using the BCA protein assay kit (Angle Gene Bioengineering Co., Ltd., Nanjing, China). In addition, western blot analysis was performed per treatment condition, using 15 mg of protein. Primary (anti-Nrf2, Abcam, ab31163) and secondary (Thermo Pierce, 31210) antibodies diluted at the ratio of 1 : 1000 were used for western blot analysis and were detected using SuperSignal^®^ West Dura Extended Duration Substrate. BandScan 5.0 software was used to analyse the optical density of band values.

### 2.9. Statistical Analysis

All statistical analyses were performed with SPSS version 22.0. Data were expressed as the mean ± standard deviation. Comparisons among all groups were performed with one-way ANOVA. When a significant treatment effect was detected, the least significant difference test was used to separate the means. In cases where the homogeneity of variance assumption was not satisfied, Dunnett's test was performed. *P* values <0.05 were considered indicative of a statistically significant finding.

## 3. Results

The vital signs of the control group rats were within normal values. In the OA model group, the rat fur was dull, and movement was retarded. In both MF treatment groups, these symptoms were present to a lesser extent than in the model group. There was no significant difference in weight gain or the seminal vesicle indices among groups (*P* > 0.05, Table 2). One rat in each of the OA model group and MF treatment groups died during the experiment. And 9 rats in each of these groups were included for analysis in our study. The testis index value was lower in the OA model group than in the control group (*P* < 0.05). The testis index values in both MF treatment groups were higher than those in the model group (*P* < 0.01).

GTW reduced both sperm count and motility in the epididymis. Sperm count and motility were higher in both MF treatment groups than in the model group (*P* < 0.05 or 0.01). No significant difference in these parameters was observed between the high-dose MF and control groups (*P* > 0.05). Sperm motility was significantly higher in the high-dose MF group than in the low-dose MF group (*P* < 0.01, Table 3).

**Figure 1:**
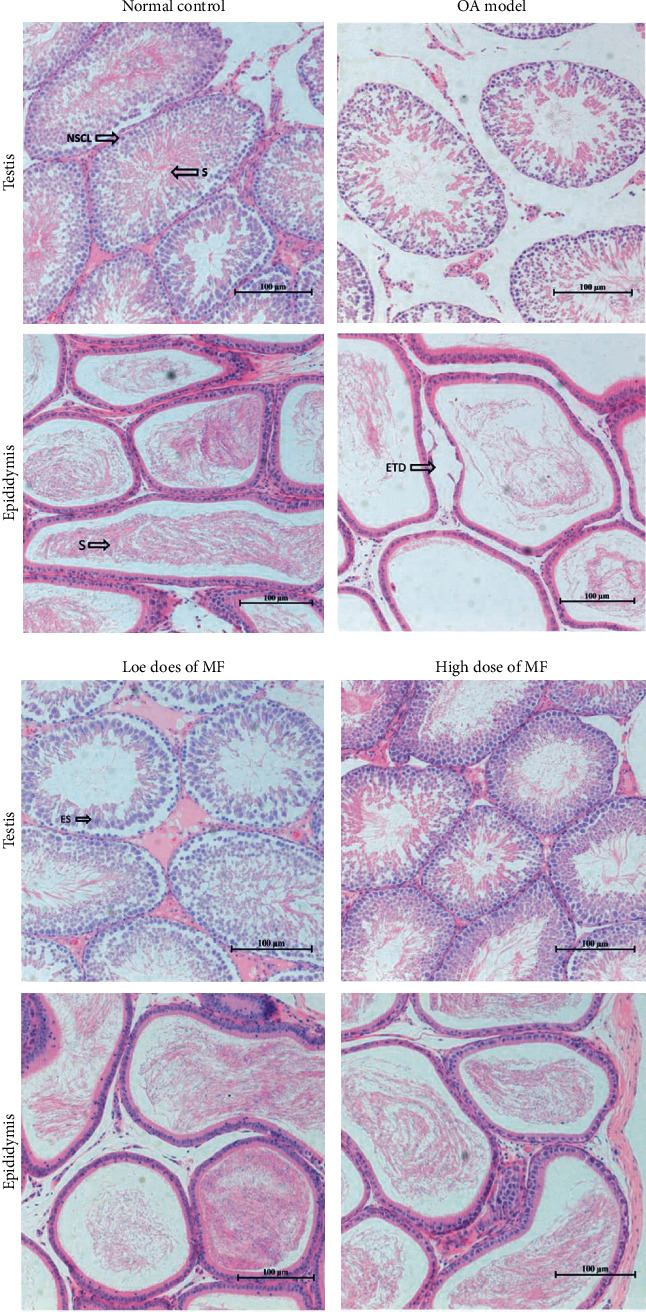
Effects of MF on the histopathological appearance of the testes and epididymis in the rat OA model. NSCL: normal spermatogenic cell line; S: sperm; ES: elongated spermatogonia; ETD: epididymis tubule detachment. Haematoxylin and eosin (H&E) staining, ×200 magnification.

In the control group, the spermatogenic cells in the seminiferous tubules were arranged in an orderly manner; the spermatogenic cells at different development levels were distinct, and a large amount of sperm was observed in the spermatophore. In the epididymis, a large number of spermatozoa were present; no nonsperm cells were observed (Figure 1).

The number of spermatozoa in the seminiferous tubules was significantly reduced; the spermatogenic cells in the seminiferous tubules were shedding and were arrayed in the OA model group, where the gap between the epididymis tubules was widened.

**Figure 2:**
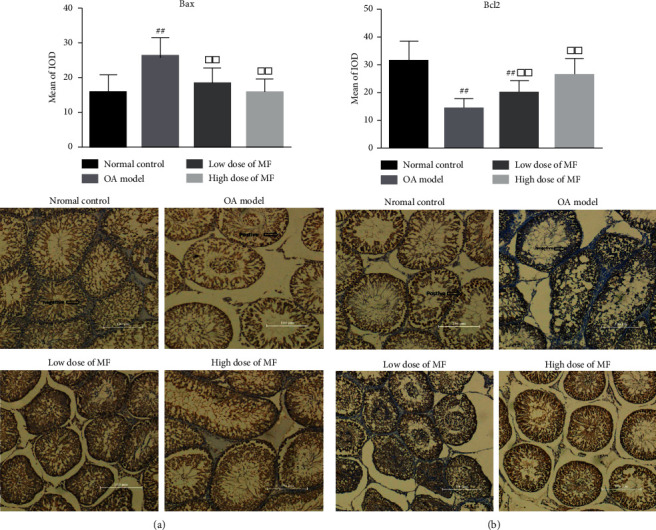
Expression levels of Bax and Bcl2 in testicular tissues. Findings from immunohistochemical staining. Bax and Bcl2 expression was indicated by brown colour in the cytoplasm (200x).

In the low-dose MF group, the spermatogenic cells in the testis of rats were arranged in a relatively orderly manner; the sperm count in the seminiferous tubules of this group was higher than that of the model group. Partially exfoliated spermatogenic cells remained visible in the seminiferous tubules. Mature sperm count was higher in this than in the model group, but lower than that in the high-dose MF group. The number of sperm in the epididymis began to recover; however, nonsperm cell components remained visible; the amount of sperm in some lumen was reduced. Histomorphology findings of the high-dose MF group testis were similar to those of the control group. The spermatogenic cell was rich in the spermatogenic tubule; higher mature sperm counts were observed in the lumen than elsewhere, and spermatogenic cells were arranged in order; finally, more sperm were found in the epididymis. Compared with the control group, the expression of Bax in the testicular tissue of the model group was significantly upregulated; in contrast, the expression of Bcl2 was downregulated (*P* < 0.01, Figure 2).

MF treatment was associated with decreased and increased expression levels of Bax and Bcl2, respectively, compared to those observed in the model group (*P* < 0.01, Figure 2). The expression levels of antioxidant indexes (SOD, CAT, MDA, and TAC) in the model group were different from those in the control group **(**Table 4**)**. In both treatment groups, the levels of SOD, CAT, MDA, and TAC were significantly improved, in particular, in the high-dose MF group (*P* < 0.05 or 0.01).

**Figure 3:**
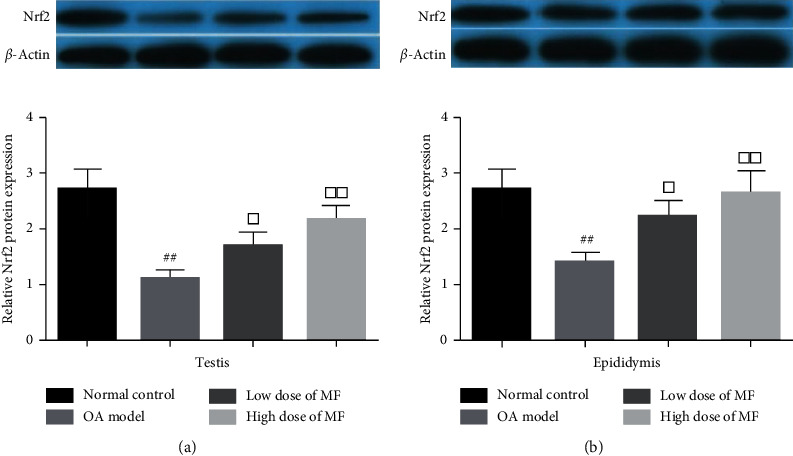
Western blot analysis of the Nrf2 protein expression in the rat testicular and epididymis tissues. Data are presented as the mean ± standard deviation. LMF: low-dose MF; HMF: high-dose MF. ^#^*P* < 0.05 and ^##^*P* < 0.01, for comparisons with the control group; ^□^*P* < 0.05 and ^□□^*P* < 0.01, for comparisons with the model group; ^△^*P* < 0.05 and ^△△^*P* < 0.01, for comparisons with the LMF group.

In the OA model group, the expression levels of Nrf2 were notably lower than those in the control group (*P* < 0.01, Figure 3). However, the levels of Nrf2 were markedly increased in the MF treatment groups, in particular, in the high-dose MF group (*P* < 0.01, Figure 3).

## 4. Discussion

It has been estimated that 50 million men worldwide are affected by infertility [35]. There is currently no treatment for patients with idiopathic OA [3, 4], although it is urgently required. Spermatogenic cell apoptosis and oxidative stress have been proposed as candidate mechanisms. The present study aimed to examine the protective effects of MF on GTW-induced reproductive system injury in a rat model.

The model of OA may be assessed based on the quality of sperm. In the present study, sperm count and motility were both reduced relative to baseline in the model rats, suggesting the model was established. Model rats were subsequently treated with high and low dose of MF; an increase in both sperm count and motility was observed in both treatment groups; the magnitude of these effects was greater in the high-dose group than in the low-dose group, suggesting protective effects of MF on the GTW-induced OA in rats.

Changes to weight and the viscera index values may reflect changes to the overall condition of the rats, including organ damage. In the present experiment, GTW did not affect either of these parameters; in contrast, testicular index values decreased significantly, suggesting that GTW may cause testicular damage. This finding is similar to that previously reported by Mei et al. in a study that involved 28 days of continuous gavage of GTW at the dose of 30 mg (kg bw)^−1^ [36]. In MF treatment groups, testicular index values increased, suggesting that MF may reverse testicular damage caused by GTW. Moreover, histomorphology findings suggest that GTW may induce the atrophy of contorted seminiferous tubules and thinning of the seminiferous epithelium, reducing the number of spermatogenic cells. MF may reverse the damage to the testis and epididymis.

The Bax and Bcl2 expression promotes and inhibits apoptosis, respectively [37]; apoptosis is determined by the ratio of these proteins. In the present study, in the GTW model group, the expression of Bax and Bcl2 in the rat testis was higher and lower, respectively, than in the control group, suggesting GTW may promote the apoptosis of spermatogenic cells, inhibiting spermatogenesis. Meanwhile, the administration of MF significantly reduced the ratio of Bax/Bcl2, suggesting that MF may reverse the apoptotic effects of GTW, protecting spermatogenesis.

Sperm is particularly sensitive to oxidative damage. Most of its cytoplasm is discarded in the final stage of sperm formation, including a group of antioxidant enzymes. The sperm membrane contains a large amount of unsaturated fatty acids, which are particularly sensitive to free radical damage. Oxygen free radicals can cause oxidative sperm damage, reducing sperm motility, which may lead to fertility decline or loss [38]. Based on this evidence, antioxidation is among the leading themes of research into infertility treatment. In the present study, the activity of SOD and CAT in the testis and epididymis of GTW model rats differed from that observed in the control group, suggesting that GTW may have an oxidative effect on the testis and epididymis of rats. Treatment with MF improved testicular and epididymal SOD and CAT activity, MDA content, and testicular TAC levels, relative to those observed in the model rats.

The expression of Nrf2 in the testis and epididymis of the GTW model rats was significantly lower than that of the control rats. The expression level of Nrf2 in both treatment groups was significantly higher than that in the model rats. GTW may cause oxidative damage to rat testis and epididymis, while MF supplementation may improve the levels of antioxidants and Nrf2 expression in the testis and epididymis. Nrf2 is part of the endogenous antioxidant signalling pathway, specifically, the Kelch-like epichlorohydrin-related protein-1 (epoxy chloropropane Kelch sample related protein-1, Keap1)-Nrf2/antioxidant response (antioxidant response element, ARE) signalling pathway [39]. This pathway is resistant to oxidative stress and contributes to the body's response to external damage; it is considered the body's most important endogenous antioxidant pathway and remains a research focus [40].

The Nrf2 protein belongs to the family of CNC regulatory proteins and has a basic leucine zipper structure, which has been observed in various body organs, where it regulates cellular redox reactions [41]. Under physiological conditions, Nrf2 binds to Keap1 and remains in an inactive state in the cytoplasm and rapidly degrades when exposed to ubiquitin-proteasome. Stimulated by reactive oxygen species or other nucleophiles, Nrf2 uncouples from Keap1 and enters the nucleus, where it binds to the Maf protein and forms a heterodimer that binds to ARE, activating target gene expression and regulating antioxidant enzyme activity. Transcriptional activity plays an antioxidant role [42, 43]. Wang et al. previously showed that GTW can cause the translocation of Nrf2 from the cytoplasm to the nucleus, resulting in large-scale hydrolysis of Nrf2 in the cytoplasm by ubiquitin hydrolase, which disrupts the Nrf2-mediated antioxidant pathway in vivo [44]. These mechanisms may account for the GTW-associated decrease in the expression level of Nrf2 protein in vivo; however, the details of this mechanism require further research. MF can increase the expression of Nrf2 protein in vivo, suggesting that it may regulate the antioxidant enzyme activity and increase the body's antioxidant capacity by regulating the Keap1-Nrf2/ARE signalling pathway; this mechanism may account for the antioxidant capacity of MF.

## 5. Conclusions

In summary, the present study has demonstrated that MF may protect spermatogenesis from the damage associated with GTW. The present measures of sperm count and motility and histopathological and immunohistochemical findings suggest that MF may help reverse GTW-related OA. In addition, the present findings regarding oxidative and antioxidant indexes (SOD, CAT, MDA, and TAC) suggest the possible mechanism underlying MF, which may involve Nrf2 mediation. The present findings suggest that MF may improve OA and inhibit the apoptosis of spermatogenic cells. The therapeutic effect of 200 mg (kg bw)^−1^ of MF is superior to that of 100 mg (kg bw)^−1^. Previous studies on MF synthesis are scarce, and its mechanisms of action are unclear. This study provides novel evidence on the therapeutic effects of MF in the treatment of OA in animal models. The involvement of Bax/Bcl2 and Nrf2 expression may account for the MF mechanism of action. The limitation of this study is that it did not account for Nrf2 expression and distribution within the cell. The present evidence should be considered preliminary; further studies are required.

## Figures and Tables

**Table 1:** Composition of each 350 mg Moriamin Forte capsule with details of each component's content.

EAAs		Vitamins		Other ingredients	
L-leucine	18.3 mg	Vitamin A	2,000 IU	5-Hydroxyanthranilic acid hydrochloride	0.2 mg
L-isoleucine	5.9 mg	Vitamin B1 nitrate	5.0 mg	—	—
L-lysine hydrochloride	25.0 mg	Vitamin B2	3.0 mg	—	—
L-phenylalanine	5.0 mg	Nicotinamide	20.0 mg	—	—
L-threonine	4.2 mg	Vitamin B6	2.5 mg	—	—
L-valine	6.7 mg	Folic acid	0.2 mg	—	—
L-tryptophan	5.0 mg	Calcium pantothenate	5.0 mg	—	—
L-methionine	18.4 mg	Vitamin B12	1.0 *μ*g	—	—
—	—	Vitamin C	20.0 mg	—	—
—	—	Vitamin D2	200 IU	—	—
—	—	Vitamin E	1.0 mg	—	—

**Table 2:** Weight gain and viscera indices of present study rats.

	Control (*n* = 10)	Model (*n* = 9)	LMF (*n* = 9)	HMF (*n* = 9)
Weight gained (g)	224.00 ± 21.16	199.89 ± 22.65	213.22 ± 38.25	203.78 ± 26.14
Testis index (%)	0.93 ± 0.07	0.83 ± 0.07^#^	0.97 ± 0.10^□□^	0.96 ± 0.45^□□^
Epididymis index (%)	0.18 ± 0.03	0.20 ± 0.03	0.21 ± 0.04	0.20 ± 0.02
Seminal vesicle index (%)	0.27 ± 0.05	0.25 ± 0.06	0.31 ± 0.08	0.28 ± 0.02

Data are presented as the mean ± standard deviation. LMF: low-dose MF; HMF: high-dose MF. ^#^*P* < 0.05 and ^##^*P* < 0.01, for comparisons with the control group; ^□^*P* < 0.05 and ^□□^*P* < 0.01, for comparisons with the model group.

**Table 3:** Sperm count and motility rates in male study rats.

	Control (*n* = 10)	Model (*n* = 9)	LMF (*n* = 9)	HMF (*n* = 9)
Cauda sperm count (×10^7^/g)	40.61 ± 8.18	25.51 ± 3.64^##^	33.22 ± 10.90^□#^	36.26 ± 6.52^□□^
Sperm motility (%)	70.50 ± 7.62	36.78 ± 9.47^##^	57.00 ± 4.64^##□□^	71.33 ± 5.52^□□△△^

Data are presented as the mean ± standard deviation. LMF: low-dose MF; HMF: high-dose MF. ^#^*P* < 0.05 and ^##^*P* < 0.01, for comparisons with the control group; ^□^*P* < 0.05 and ^□□^*P* < 0.01, for comparisons with the model group; ^△^*P* < 0.05 and ^△△^*P* < 0.01, for comparisons with the LMF group.

**Table 4:** Levels of SOD and CAT activity, MDA concentration, and TAC in rat testicular and epididymal tissues.

	—	Control (*n* = 10)	Model (*n* = 9)	LMF (*n* = 9)	HMF (*n* = 9)
Testis	SOD (U/mg)	51.55 ± 4.24	33.60 ± 2.78^##^	43.47 ± 5.91^#□□^	51.49 ± 2.57^□□△^
	CAT (U/mg)	72.49 ± 6.92	58.13 ± 7.69^##^	61.80 ± 8.5^#^	66.99 ± 11.72^□^
	MDA (nmol/mg)	1.21 ± 0.26	1.53 ± 0.35^#^	1.23 ± 0.36^□^	1.08 ± 0.25^□□^
	TAC (U/mg)	39.06 ± 6.80	22.99 ± 5.88^##^	29.10 ± 5.99^##□^	35.04 ± 5.49^□□△^

Epididymis	SOD (U/mg)	46.30 ± 3.02	34.76 ± 2.82^##^	37.93 ± 2.80^##□^	45.93 ± 3.14^□□△△^
	CAT (U/mg)	64.56 ± 8.76	49.46 ± 8.93^##^	57.80 ± 9.20^□^	61.05 ± 5.00^□□^
	MDA (nmol/mg)	1.09 ± 0.30	1.52 ± 0.34^##^	1.16 ± 0.33^□^	1.06 ± 0.21^□□^
	TAC (U/mg)	33.18 ± 7.19	19.86 ± 4.84^##^	25.34 ± 4.68^##^	32.75 ± 7.58^□□△^

Data are presented as the mean ± standard deviation. LMF: low-dose MF; HMF: high-dose MF. ^#^*P* < 0.05 and ^##^*P* < 0.01, for comparisons with the control group; ^□^*P* < 0.05 and ^□□^*P* < 0.01, for comparisons with the model group; ^△^*P* < 0.05 and ^△△^*P* < 0.01, for comparisons with the LMF group.

## Data Availability

The data used to support the findings of this study are available from the corresponding author upon request.
